# Chitosan Aerogel Particles as Nasal Drug Delivery Systems

**DOI:** 10.3390/gels8120796

**Published:** 2022-12-04

**Authors:** Natalia Menshutina, Alexander Majouga, Anastasia Uvarova, Daria Lovskaya, Pavel Tsygankov, Maria Mochalova, Olga Abramova, Valeria Ushakova, Anna Morozova, Artemiy Silantyev

**Affiliations:** 1 Department of Chemical and Pharmaceutical Engineering, Mendeleev University of Chemical Technology, Moscow 125047, Russia; 2 Department of Basic and Applied Neurobiology, V. Serbsky Federal Medical Research Centre of Psychiatry and Narcology, Kropotkinsky per. 23, Moscow 119034, Russia

**Keywords:** aerogel, chitosan, spraying, homogenization, particles, drug delivery systems, pharmacokinetics, in vivo experiments

## Abstract

The nasal drug delivery route has distinct advantages, such as high bioavailability, a rapid therapeutic effect, non-invasiveness, and ease of administration. This article presents the results of a study of the processes for obtaining chitosan aerogel particles that are promising as nasal or inhalation drug delivery systems. Obtaining chitosan aerogel particles includes the following steps: the preparation of a chitosan solution, gelation, solvent replacement, and supercritical drying. Particles of chitosan gels were obtained by spraying and homogenization. The produced chitosan aerogel particles had specific surface areas of up to 254 m^2^/g, pore volumes of up to 1.53 cm^3^/g, and porosities of up to 99%. The aerodynamic diameters of the obtained chitosan aerogel particles were calculated, the values of which ranged from 13 to 59 µm. According to the calculation results, a CS1 sample was used as a matrix for obtaining the pharmaceutical composition “chitosan aerogel—clomipramine”. X-ray diffraction (XRD) analysis of the pharmaceutical composition determined the presence of clomipramine, predominantly in an amorphous form. Analysis of the high-performance liquid chromatography (HPLC) data showed that the mass loading of clomipramine was 35%. Experiments in vivo demonstrated the effectiveness of the pharmaceutical composition “chitosan aerogel—clomipramine” as carrier matrices for the targeted delivery of clomipramine by the “Nose-to-brain” mechanism of nasal administration. The maximum concentration of clomipramine in the frontal cortex and hippocampus was reached 30 min after administration.

## 1. Introduction

The development of nasal drug delivery systems is a complex and promising area of modern science due to their advantages. Nasal delivery systems provide a rapid therapeutic effect, high drug stability, improved physicochemical properties (water solubility and bioavailability), and the masking of taste or smell [1,2]. These advantages are associated with the high permeability of the nasal mucosa and the extensive vascular system of the nasal cavity [3]. Nasal drug delivery systems are suitable for both topical and systemic use.

Many articles describe the preparation of nasal delivery systems in the form of emulsions, sprays, liposomes, in situ gel systems, microemulsions, nanoemulsions [2], and microparticles obtained using the emulsion polymerization technique, the spray drying method, and lyophilization [3,4,5].

Dry nasal delivery systems, such as microparticles, delay mucociliary clearance, thereby increasing the contact time between the drug delivery system and the mucosa compared to liquid formulations [1,3]. This article presents the development of dry nasal drug delivery systems based on biopolymer aerogels.

Biopolymer aerogels are highly porous materials with high surface area (up to 500 m^2^/g) and porosity (up to 99%), as well as low density (up to 0.5 g/cm^3^) [6,7]. Supercritical drying (SCD) technology is used to preserve the nanoporous structure of the gel and prevent pore collapse, protecting the dried aerogels from cracking and shrinkage [8].

Currently, the production processes and subsequent analysis of the structure, the search for relationships between the type of polysaccharide used (agar-agar, alginate, cellulose, pectin, lignin, starch, protein, chitosan, etc.), the parameters of the production process, and the final properties of aerogels are widely studied [9]. Biodegradability and biocompatibility are the distinguishing properties of polysaccharide-based aerogels [10]. It is these qualities that make them promising materials for use in various fields of science. The material that forms the solid framework of the aerogel determines the chemical affinity for various drugs, which, in turn, defines the loading efficiency and the release pattern of active substances [11]. Therefore, such a variety of initial materials used to produce aerogels allows selecting the compositions of the “aerogel—active substance” for the development of a drug delivery system with the desired final properties of the matrix.

Chitosan is a natural-origin linear polysaccharide usually derived from chitin by alkaline deacetylation [12,13]. Chitin is the second most abundant polysaccharide [12]. Chitosan is a permeability enhancer, mucoadhesive, and is biocompatible [12,14]. The structure of chitosan contains hydroxyl and amino groups, which allow chitosan to form hydrogen and covalent bonds. The positive charge of chitosan provides ion–ion interaction between the molecule and the negatively charged surface of the nasal mucosa, which leads to a mucoadhesive effect [14]. A study of the composition “chitosan powder—insulin” was carried out on sheep [15]. The bioavailability of insulin increased by 20%. These properties make chitosan aerogels promising nasal, inhalation, and oral delivery systems for active substances.

The shape of the resulting aerogels can be different, but biopolymer aerogels in the form of particles are the most promising for applications in medicine, tissue and regenerative medicine, pharmaceuticals, and the food industry [9,10,16,17]. The introduction of active pharmaceutical ingredients (APIs), enzymes, and vitamins into the aerogel matrix makes it possible to produce “aerogel—active substance” compositions for use as drug delivery systems or scaffolds for tissue engineering and wound dressing [10,17,18,19]. In the study [20], it was shown that the loading of active substances in aerogel was higher in spherical particles than in monoliths. The use of particulate chitosan aerogels as a hemostatic agent has been investigated [17]. It was shown that primary hemostasis was achieved in all in vivo experiments on laboratory animals using chitosan aerogel particles.

The main stages of obtaining aerogel particles based on polysaccharides are: the preparation of the initial polymer solution, formation of solution droplets by various methods, gelation, solvent replacement, and SCD [21].

There are two main groups of methods for forming drops of a solution [9].

1. Spraying. Drops of a polysaccharide solution are formed in a gaseous environment. After that, they fall into a container with a solution of a cross-linking agent, which, upon contact with, gelation occurs.
2. Homogenization. Drops of the polysaccharide solution are formed in the liquid phase. An emulsion of a solution of a polysaccharide in oil is formed during stirring and undergoes further gelation.

SCD is the only approach to obtaining aerogels. The peculiarity of this technology is that during the drying process, there is no “gas-liquid” phase boundary in the pores of the material. This allows the preservation of the developed structure of gels [1]. Thus, it is possible to obtain aerogels with a high specific surface area.

Drying in a supercritical fluid environment is a clean and environmentally friendly process that complies with the requirements of “green chemistry”. This technology can be scaled up with the conditions specified in the protocols of good manufacturing practice [11]. The use of carbon dioxide, CO_2_, compared to other supercritical fluids (water, nitric oxide, ethane, or propane) is safe for processing pharmaceutical and food products [11]. The advantages of using CO_2_ include low toxicity and environmental friendliness, non-combustibility, and the possibility of recovery. Supercritical conditions are achieved at moderate pressure (73 bar) and temperature (31.1 °C), making the process more economical than other supercritical fluids [22]. The low temperature also allows the processing of thermolabile substances.

Over the past few years, both organic and inorganic aerogels have shown great promise as drug delivery systems. The large specific surface area of aerogels contributes to the ability to load and release bioactive agents, such as APIs or antibiotics [23]. Various methods are used to load active substances into aerogels. The choice of the method is due to the physical and chemical properties of the used active substances. There are several ways to introduce active substances into the pores of the aerogel. The substance can be loaded at the stage of preparing a polymer solution [24], at the stage of washing [20], at the last stage of solvent replacement [8,25], during supercritical drying, or into an already prepared aerogel, using supercritical adsorption or adsorption from a solution [19,25,26,27].

The preparation of pharmaceutical compositions based on alginate aerogels with loaded ketoprofen, nimesulide, and loratadine, using supercritical adsorption, was described [19]. During the study of the dissolution test (in vitro dissolution tests), the rate of release and dissolution of the API increased several times. Thus, the authors noted that the preparation of pharmaceutical compositions made it possible to reduce the time required for the release and dissolution of 50% API up to 6.6 times. Improved pharmacokinetic properties would lead to a faster therapeutic effect.

Various approaches can be used to overcome the blood–brain barrier (BBB) [28,29]. One of these approaches is nasal administration. Aerogel particles may be useful as nasal drug delivery systems. The advantages of the nasal delivery route include non-invasiveness, ease of use, high bioavailability, rapid onset of the therapeutic effect, and reduced side effects [30,31]. The main feature of nasal delivery is delivery by the “Nose-to-Brain” mechanism, which allows drugs to be delivered to the central nervous system (CNS) through the olfactory and trigeminal nerves, bypassing the BBB [12,31,32]. This delivery method can be effective in the treatment of diseases such as Alzheimer’s disease, Parkinson’s disease, migraines, cardiovascular diseases, for pain relief, or in emergency situations, for example, epilepsy [31,33,34]. The distinguishing advantages of drug delivery systems based on chitosan aerogels include high mucoadhesive properties and high permeability [35].

The study [36] showed that the optimal particle size of nasal dry sprays to ensure the greatest adhesion to the mucous membrane was a diameter of 50–100 µm. It was noted that particles with a diameter of less than 20 microns can provide delivery to the lungs, so such particles can be used to create inhaled drug delivery systems.

Based on the reviewed articles, it was concluded that pharmaceutical compositions based on chitosan aerogels in the form of particles, as well as the study of their effectiveness, are a perspective, promising, and relevant research topic. The main purpose of this work was to obtain chitosan aerogel particles by spraying and homogenization methods for use as nasal drug delivery systems and to evaluate the effectiveness of the pharmaceutical composition “chitosan aerogel—clomipramine” for nasal administration on a model of depressive-like state in rats.

## 2. Results and Discussion

### 2.1. Study of the Internal Structure of Chitosan Aerogel Particles

**Figure 1:**
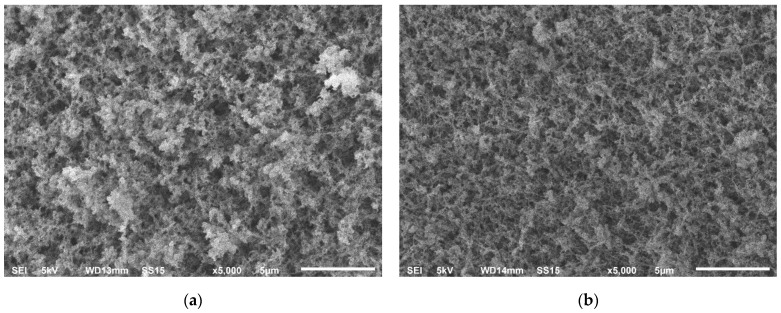
SEM images of the inner surface of chitosan aerogel particles: (**a**) CS1 sample obtained by spraying; (**b**) CH1 sample obtained by homogenization.

SEM images of the inner surface of chitosan aerogel particles are shown in Figure 1.

The morphology of chitosan aerogel particles obtained by spraying and homogenization was represented by a developed globular fibrous structure. Based on the data, the dispersion method did not affect the inner surface of the samples at the selected preparation parameters.

Table 1 lists the main characteristics used in describing the structure of the aerogels: specific surface area (S), average pore volume (V_BJH_), pore diameter (D_pore_), skeletal density (p_skeletal_), bulk density (p_bulk_), porosity (P), average particle size (d_av_), and mean aerodynamic diameter (d_aero_).

Analytical studies showed that all samples were mesoporous materials, since the value of the average pore diameter lay in the range of 24–26 nm. It can be concluded that the materials were obtained with similar values of characteristics: specific surface area varied from 212 to 254 m^2^/g, and the average pore volume ranged from 1.21 to 1.51 cm^3^/g. It should be noted that the porosity of the samples reached 99%.

Since chitosan aerogel particles are highly porous materials, the aerodynamic diameter was additionally estimated. The aerodynamic diameter means a diameter of a sphere with a density of 1 g/cm^3^, which has the same settling velocity as a considered particle, due to the force of gravity [37]. Thus, the concept of aerodynamic diameter successfully describes the aerodynamic behavior of particles in air and in many other systems, considering the shape and density of particles.

Gel particles with an average diameter of 130 to 300 μm and an average aerodynamic diameter of 22 to 59 μm were obtained by spraying, using a Glatt GmbH nozzle with an inner diameter of 0.5 mm and an overpressure of 0.1 bar applied to the nozzle. Gel particles with an average diameter of less than 95 µm and an aerodynamic diameter of less than 26 µm were obtained by homogenization on an IKA T25 ULTRA-TURRAX at a homogenization speed of 6000 rpm.

Obtained chitosan aerogel particles can be used for various purposes. For example, aerogel particles obtained by spraying with the described parameters can be used to create nasal delivery systems, since their aerodynamic characteristics allow them to be deposited in the nasal cavity. Aerogel particles obtained by the homogenization method with the selected parameters have a smaller aerodynamic diameter and therefore can be used for inhalation delivery [35,36].

For example, these materials can be used as drug delivery systems. Currently, the number of articles describing the successful introduction of active substances, such as APIs, vitamins, antibiotics, and vaccines, into aerogels is increasing [11,19,35,38]. The study [39] showed that surface area, average pore volume, and pore diameter affect the amount of API mass loading during the preparation of compositions. Large values of specific surface areas allow more APIs to be adsorbed inside the pores. The presence of a large number of mesopores (2–50 nm) in the aerogel structure presumably minimizes the appearance of crystalline forms of APIs [19,38]. Therefore, it is possible to obtain APIs predominantly in the amorphous state, which improves the pharmacokinetic properties of the pharmaceutical composition “aerogel—API”, since the dissolution of substances in the amorphous state does not require energy and time to destroy the crystal lattice [19].

### 2.2. The Results of Measuring the Amount of Adsorbed Substance by HPLC

A calibration line for known concentration solutions of clomipramine was constructed. The amount of adsorbed API was further determined from this calibration line.

Sample preparation was carried out for the obtained sample. A weighed portion of aerogel with loaded API was dissolved in the mobile phase. The sample was then filtered and detected three times. The API concentration was recalculated according to the obtained equation of the calibration line. The mass loading of the API into the aerogel matrix was calculated using the formula:

(1) $$\omega =\frac{m_{\mathrm{API}}}{m_{\mathrm{PC}}}\times 100\%,$$

where $m_{\mathrm{API}}$ is the API mass calculated according to the equation of the calibration curve, g, and $m_{\mathrm{PC}}$ is the mass of the dissolved pharmaceutical composition, g.

The average mass loading was determined as the arithmetic average of three measurements.

**Figure 2:**
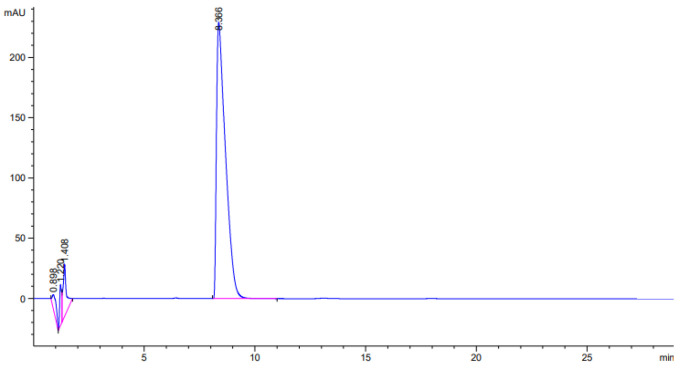
Chromatogram of clomipramine loaded in chitosan aerogel.

The chromatogram of the pharmaceutical composition “chitosan aerogel—clomipramine” is shown in Figure 2.

The results of the analysis showed that the mass loading of clomipramine in the CS1 sample was 35 wt.%. Thus, obtaining the pharmaceutical composition by adsorption from the solution is an effective way to load the API.

### 2.3. The Results of the Study by XRD Analysis of the Pharmaceutical Composition “Chitosan Aerogel—Clomipramine”

**Figure 3:**
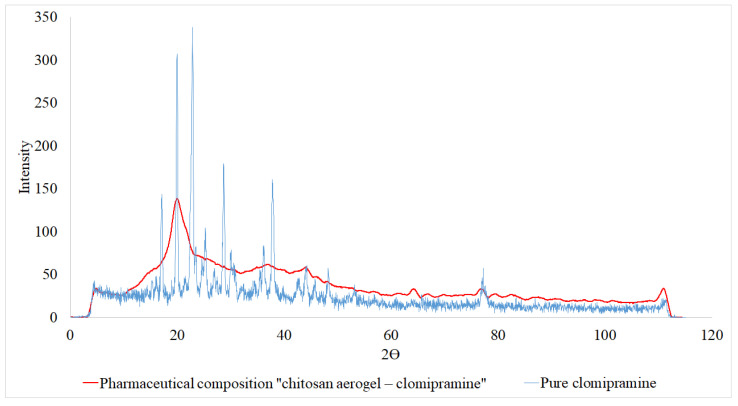
Diffractograms of pure clomipramine and pharmaceutical composition “chitosan aerogel—clomipramine”.

Diffractograms of the sample were obtained using the method of XRD analysis. The analysis was carried out using an InelEquinox 2000 X-ray powder diffractometer. Diffractograms of pure clomipramine and the pharmaceutical composition “chitosan aerogel—clomipramine” are shown in Figure 3.

The peaks corresponding to the crystalline state of clomipramine are absent in the diffractogram of the pharmaceutical composition “chitosan aerogel—clomipramine”. Thus, it can be concluded that clomipramine is predominantly in the amorphous state in chitosan aerogel particles. This can provide an improvement in its properties, such as solubility (less energy is spent on the destruction of the amorphous structure than on the destruction of the crystalline form) and stability (the API cannot be oxidized longer due to being in an amorphous form). There are studies in the literature that reflect an increase in the solubility kinetics of drugs included in aerogels [19,40]. 

Therefore, studies on rats were carried out to determine the effectiveness of the obtained pharmaceutical composition “chitosan aerogel—clomipramine”.

### 2.4. Animal Experiments

The animal experiment was performed to study the accumulation of clomipramine, which was adsorbed in chitosan aerogel particles, in brain regions of rats, as well as the dynamics of its elimination from the rats’ blood. In addition, the effect of chronic administration of clomipramine in a depression-like state model was evaluated. The experiments were performed at the National Medical Research Centre of Psychiatry and Narcology, named after V.P. Serbsky.

#### 2.4.1. Behavioral Experiment

**Figure 4:**
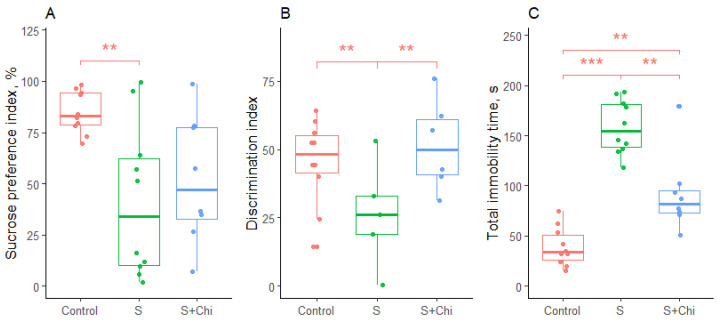
The results of testing the behavior of rats in the sucrose preference test (**A**), new object recognition test (**B**), and forced swim test (**C**). Control—control group, S—chronic stress group, and S+Chi—group of rats exposed to chronic stress with intranasal treatment. **—*p* < 0.01; ***—*p* < 0.001.

Statistically significant differences were shown in a sucrose preference test (F = 6.4; *p* = 0.006), new object recognition test (F = 9; *p* = 0.001), and forced swim test (χ^2^ = 21; *p* < 0.001). The results of the experiments are shown in Figure 4. 

Significant differences between groups were demonstrated by the new object recognition test (F = 9; *p* = 0.001). Chronic stress lowered the discrimination index compared to the control group (*p* = 0.004). However, this disorder was completely neutralized with the pharmaceutical composition “chitosan aerogel—clomipramine” therapy. The discrimination index in the therapy group recovered to the control level and significantly differed from the stress group (*p* = 0.003) (Figure 4B).

Finally, significant differences were observed in the forced swim test (χ^2^ = 21; *p* < 0.001). Chronic stress increased the level of immobility in rats compared to the control group (*p* < 0.001). However, the therapy reduced the level of immobility compared to the stress group (*p* = 0.009), but the animals in the treatment group still froze more than those in the control group (*p* = 0.005) (Figure 4C).

#### 2.4.2. Distribution of Drugs in Blood and Brain Tissue

**Figure 5:**
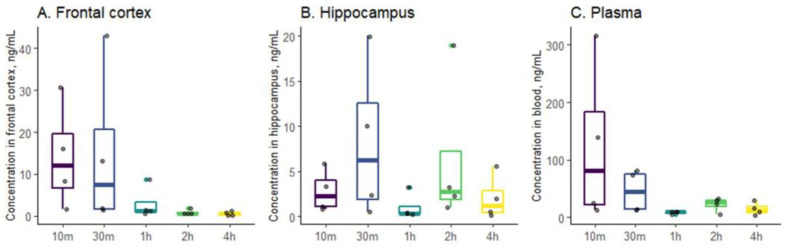
Distribution of clomipramine in frontal cortex (**A**), hippocampus (**B**), and blood plasma (**C**) of rats.

Different regularity was observed when the pharmaceutical composition “chitosan aerogel—clomipramine” was administered intranasally to rats. The concentration of clomipramine decreased already after 1 h to minimum detectable values in the frontal cortex, hippocampus, and plasma (Figure 5). However, when the pharmaceutical composition “chitosan aerogel—clomipramine” was administered, this ratio was approximately 6 for the hippocampus and 30 for the frontal cortex.

The maximum concentration of clomipramine in the hippocampus and in the frontal cortex was reached 30 min after intranasal administration of the composition. The peak concentration of the API was observed in plasma after 10 min. Thus, the use of this composition allows quickly and efficiently delivering API to the brain and blood, which can contribute to a rapid therapeutic effect.

## 3. Conclusions

The study of the processes of obtaining organic aerogels based on chitosan in the form of particles by various methods, spraying and homogenization, was carried out in this work. The results of experimental and analytical studies showed that gel particles with an average diameter of 200 µm, by the spray method, and 90 µm by the oil-emulsion method, were obtained. It was shown that by using these methods for obtaining gel particles, it is possible to obtain aerogels with a wide range of characteristics. These characteristics can be controlled and varied through process parameters depending on the end use of the material.

Experiments were carried out to obtain the pharmaceutical composition “chitosan aerogel-clomipramine” for targeted delivery by the nose-to-brain mechanism for the treatment of depressive disorders. It was shown that the mass loading of clomipramine is more than 30 wt.%. The results of XRD analysis showed that the API is predominantly in the amorphous state, which increases its solubility.

Experiments in vivo on male mice were carried out. The pharmaceutical composition “chitosan aerogel-clomipramine” partially reversed behavioral responses in rats that had been impaired by chronic stress. The main test showing stress response was the forced swimming test. This is the main test for testing antidepressants. In our study, the time of immobility in rats decreased in all experiments with the use of the pharmaceutical composition “chitosan aerogel-clomipramine“, which indicates a greater resistance in rats treated with this drug to chronic stress. There was also an improvement in the cognitive functions of the rats that were impaired after the stress. Cognitive decline is one of the symptoms of depression in humans and depressive-like behavior in animals. Considering the positive results on the effect of the pharmaceutical composition “chitosan aerogel-clomipramine” on behavior, the distribution of the drug in the tissues should be investigated further. It should be noted that the method of administration should be modified.

These results demonstrate that chitosan aerogel particles are promising carrier matrices for targeted API delivery by the “Nose-to-brain” mechanism, as they have a therapeutic effect on depressed rats. Thus, the pharmaceutical composition “chitosan aerogel-clomipramine” is an effective drug for the treatment of depressive disorders.

## 4. Materials and Methods

### 4.1. Materials

In this work, acid-soluble chitosan, with a molecular weight of 111 kDa (Sigma Aldrich, Saint Louis, MO, USA), was used. Chemically pure acetic acid (99.8 wt.%, Ruschim, Moscow, Russia) was used as the acid. Pure sodium hydroxide (Ruschim, Moscow, Russia) was used as a cross-linking agent. Sunflower oil (pharmaceutical secondary standard, Sigma Aldrich, Saint Louis, MO, USA) was used to prepare the oil emulsion. Isopropyl alcohol (>99.8 wt.%, Ruschim, Moscow, Russia) was used as a solvent. The drying agent was carbon dioxide (purity 99.99%). Clomipramine hydrochloride powder (>98 wt.%, Sigma Aldrich, Saint Louis, MO, USA) was used to obtain the pharmaceutical composition “chitosan aerogel—API”. Acetonitrile (>99.5%, Sigma-Aldrich, Saint Louis, MO, USA), tetramethylethylenediamine (Sigma-Aldrich, Saint Louis, MO, USA), and triethylamine (Sigma-Aldrich, Saint Louis, MO, USA) were used to determine the amount of adsorbed substance in the aerogel matrix. Tween 80 (Sigma-Aldrich, Saint Louis, MO, USA) was used as a surfactant.

### 4.2. Preparation of Chitosan Aerogel Particles

Main stages of obtaining chitosan aerogel particles:

1. Preparation of the initial solution of chitosan in acetic acid;
2. Gelation of gel particles, based on chitosan, by various methods;
3. Step-by-step solvent replacement;
4. Supercritical drying.

**Figure 6:**
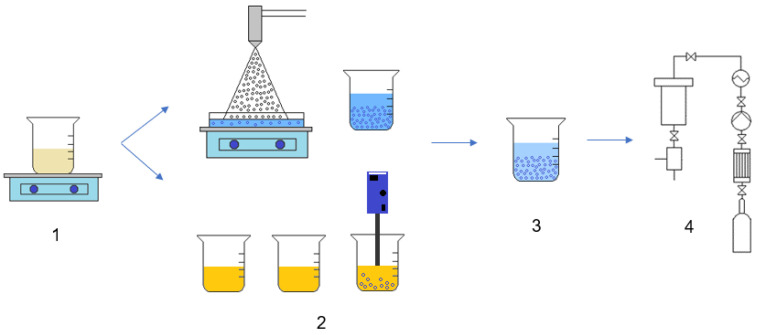
General scheme for obtaining chitosan aerogel particles: **1**—preparation of a chitosan solution, **2**—gelation stage, **3**—solvent replacement stage, and **4**—supercritical drying.

Figure 6 shows the main stages in the formation of chitosan aerogel particles.

Such parameters as the concentration of the acetic acid solution, concentration of the chitosan solution, and concentration of the sodium hydroxide solution varied during the preparation of the chitosan aerogel particles. The concentrations of the reagents during sample preparation are shown in Table 2.

The samples obtained by the spraying method were named CS1–CS4. The excess pressure of compressed air supplied to the nozzle was 0.1 bar for all experiments. In this work, a Glatt GmbH nozzle with a nozzle diameter of 0.5 mm was used.

The samples obtained by the homogenization method were designated as CH1–CH4. The volume ratio of chitosan solution to oil was 1 to 3, and the volume ratio of sodium hydroxide solution to oil was also 1 to 3 for all experiments performed. Tween 80 was used as a surfactant, similarly, in this study [41]. The use of surfactants makes it possible to reduce the surface tension of droplets formed and thereby obtain particles of a smaller diameter. This surfactant is used as a pharmaceutical excipient in drug formulation [42]. The homogenization speed was 6000 rpm in all experiments.

#### 4.2.1. Preparation of Chitosan Solution

Chitosan, in powder form, was added to an acetic acid solution with constant stirring on a magnetic stirrer at 250 rpm. The prepared solution was continuously stirred for 24 h at room temperature at a constant speed to completely dissolve the chitosan and to obtain a homogeneous solution of chitosan.

#### 4.2.2. Preparation of Chitosan Gel Particles by Spraying

**Figure 7:**
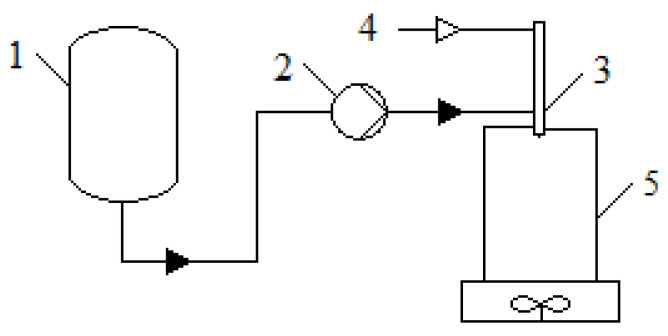
Scheme of the installation for obtaining chitosan gel particles by spraying: **1**—container with chitosan solution; **2**—peristaltic pump; **3**—nozzle; **4**—air supply line; **5**—receiving container with a stirring device.

An installation was used to implement the spraying process. A schematic diagram of the installation is shown in Figure 7.

The liquid flow rate during each experiment was selected to obtain a stable spray jet, which was necessary for fine spraying and ensuring uniform droplet formation in the solution at the selected pressure. Gelation and formation of gel particles occurred when drops fell into the sodium hydroxide solution. Stirring using a magnetic stirrer was organized in receiving container, 5, to prevent the agglomeration of the resulting particles. The mixture was kept for 24 h with slow stirring to form the final structure and ensure the completeness of the chemical reactions.

#### 4.2.3. Preparation of Chitosan Gel Particles by Homogenization 

An emulsion of a chitosan solution and an emulsion of a sodium hydroxide solution were prepared to obtain chitosan gel particles by homogenization. The emulsion of the chitosan solution in oil and the emulsion of the sodium hydroxide solution in oil were homogenized on an IKA T25 ULTRA-TURRAX at a constant speed of 6000 rpm. After that, the emulsion of the chitosan solution was added, dropwise, to the emulsion of the sodium hydroxide solution with stirring on a disperser.

The particles of the dispersed phase were formed in the process of continuous mixing. Further, an additional amount of sodium hydroxide solution was added to the reaction mixture and kept for 12 h to ensure the completeness of the chemical reactions.

#### 4.2.4. Solvent Exchange

After the gelation step, the particles were repeatedly washed with distilled water. After that, the stage of replacing the aqueous solvent with isopropyl alcohol was carried out. In this study, a stepwise solvent exchange was used (30%, 60%, 90%, 100%, and 100%). There needed to be at least 2 h between each replacement. Such a scheme was necessary so that the deformation of the gel structure did not occur. Thus, the chitosan gel particles were prepared for supercritical drying.

#### 4.2.5. Supercritical Drying

**Figure 8:**
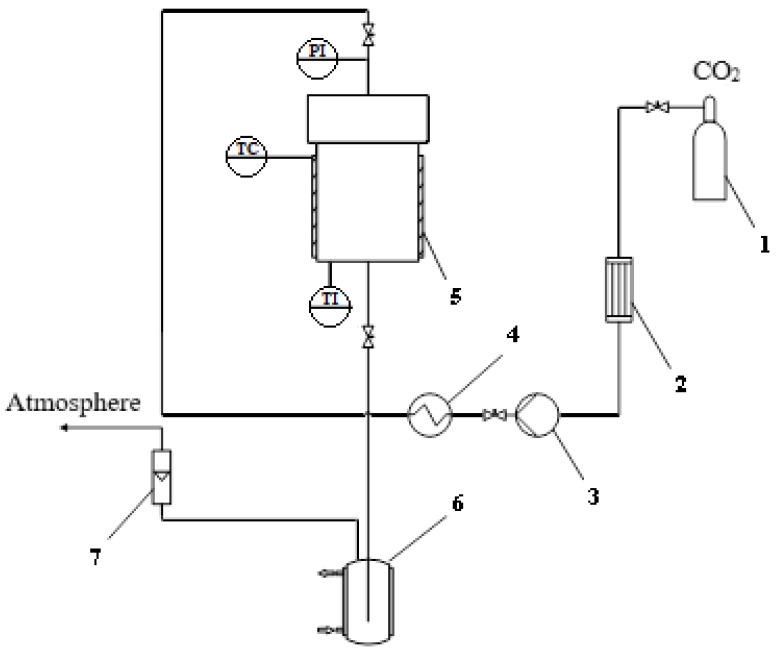
Schematic diagram of the installation for supercritical drying: **1**—cylinder with carbon dioxide; **2**—heat exchanger, **3**—high pressure piston pump, **4**—thermostat, **5**—high pressure reactor; **6**—separator with a cooling jacket; **7**—rotameter; PI—pressure gauge; TC—temperature controller; TI—temperature sensor.

Supercritical drying is the final stage in the production of chitosan aerogel particles. SCD was carried out in a high-pressure apparatus, similarly to [17,19]. The installation scheme for supercritical drying is shown in Figure 8.

Carbon dioxide (CO_2_) was supplied from a cylinder, 1, to a heat exchanger, 2. CO_2_ was cooled to 5 °C in the heat exchanger, 2, to avoid carbon dioxide phase transition. The pressure was pumped up by a pump, 3. CO_2_ entered a thermostat, 4. After which, supercritical carbon dioxide (scCO_2_) entered the high-pressure apparatus, 5. A temperature controller, manometer, and temperature sensor were installed in the high-pressure apparatus. The process of heating the apparatus came from a heating jacket attached to the body. Regulation of the carbon dioxide flow was carried out by a system of valves at the outlet of the apparatus. The consumption of gaseous carbon dioxide was displayed on a rotameter.

#### 4.2.6. Obtaining the Composition “Chitosan Aerogel—API”

Sample CS1 was used to prepare pharmaceutical compositions based on chitosan aerogel as a matrix. This sample had the largest specific surface area (254 m^2^/g) and an optimal mean aerodynamic diameter (49 µm). Clomipramine was used as the API. Clomipramine is a highly water-soluble compound [43]. Clomipramine is a tricyclic antidepressant used in the treatment of obsessive-compulsive disorder, according to the FDA [44].

The preparation of the pharmaceutical composition “chitosan aerogel—clomipramine” was carried out using the method of adsorption from a solution. Chitosan aerogel particles were contacted with a solution of clomipramine at a concentration of 150 × 10^−4^ g/mL. During the adsorption process, clomipramine diffused into the pores of the aerogel. Thus, the pharmaceutical composition “chitosan aerogel—clomipramine” was obtained.

### 4.3. Analytical Research

During this study, analytical studies, such as nitrogen porosimetry (at −196 °C) on an ASAP 2020MP specific surface and porosity analyzer, were carried out to determine the specific surface area, volume, and pore diameter. According to the results of this study, the specific surface area was determined by the Brunauer–Emmett–Teller (BET) method; pore volume and their average diameter were measured according to the Barrett–Joyner–Halenda (BDH) method. The pycnometer method was carried out on a helium pycnometer “AccuPyc II 1340” (Micromeritics) to determine the skeletal density.

The bulk density of the particles was defined as the ratio of the mass of the particles to the volume occupied and was calculated by the formula:

(2) $$p_{bulk}=\frac{m_{particles}}{V_{particles}}$$

where $p_{bulk}$ is the bulk density, g/cm^3^; $m_{particles}$ is the mass of the particles, g; and $V_{particles}$ is the volume of the particles, cm^3^. Each measurement was carried out three times. The bulk density value was determined as the arithmetic mean of three measurements.

The porosity of the chitosan aerogel particles was calculated by the formula:

(3) $$P=\left(1-\frac{p_{bulk}}{p_{skeletal}}\right)\times 100$$

where P is the sample porosity, %; $p_{bulk}$ is the bulk density, g/cm^3^; and $p_{skeletal}\;$is the skeletal density, g/cm^3^.

The laser diffraction method was used to study the particle size distribution. The analysis was carried out on an Analysette 22 Nano Tec instrument for the chitosan gel particles in distilled water. The value of the average diameters of the gel particles was calculated by the formula:

(4) $$D_{av}=\sum_{i}\frac{n_{i}}{\sum_{i}n_{i}}d_{i}=\sum_{i}f_{ni}d_{i}$$

where $D_{av}\;$is the average diameter, mk; $n_{i}\;$is the number of particles in the i-th fraction (diameter $d_{i}$); $\underset{i}{\sum }n_{i}$ is the total number of particles in the system; and *f_ni_* = *n_i_*/∑*_i_ n_i_* is the numerical fraction of the i-th fraction.

The values of the aerodynamic diameters were calculated according to the formula according to [37,45]:

(5) $$D_{aero}=\sqrt{\rho_{particle}}\times D_{av}$$

where $D_{aero}$ is the aerodynamic diameter, µm; $D_{av}$ is the average diameter, µm; and $\rho_{particle}$ is the density of the particles, g/cm^3^.

The particle density was determined through the layer porosity, ε. For particles, it is customary to consider the porosity of the layer to be 0.38–0.42 [46]. For the calculation, the average porosity was taken as equal to *ε* = 0.4. The particle density was calculated by the formula:

(6) $$\rho_{particles}=\frac{\rho_{bulk}}{1-\varepsilon }$$

where $\rho_{particles}$ is the particle density, g/cm^3^; $\rho_{bulk}$ is the bulk density, g/cm^3^; and ε is the layer porosity, cm^3^/cm^3^.

All experimental points were measured at least three times. The average results of analytical studies are presented in Table 1.

The surface morphology of the obtained particles was studied by scanning electron microscopy (SEM) on a JEOL 1610LV instrument. Studies by helium pycnometry and SEM were carried out at the Center for Collective Use of the D.I. Mendeleev Russian University of Chemical Technology.

The amount of adsorbed clomipramine in the pores of the chitosan aerogel particles was determined by high-performance liquid chromatography. This study was carried out using an Agilent 1220 Compact LC chromatograph. A method for determining the API was selected according to the literature data [47]. The mobile phase was a buffer system consisting of acetonitrile and distilled water containing 0.05 vol% tetramethylethylenediamine and 0.01 vol% triethylamine. The pH value of 6.5 was adjusted with acetic acid. Acetonitrile and the buffer system were in a ratio of 370:630. The volumetric flow rate of the mobile phase was 1.5 mL/min, the decoration wavelength was 254 nm, and the sample volume was 20 µL [47].

Qualitative XRD analysis was used to determine the presence of the crystalline or amorphous phases of clomipramine in the pharmaceutical composition “chitosan aerogel—clomipramine”. This study was carried out on an InelEquinox 2000 X-ray powder diffractometer.

### 4.4. Animal Studies

#### 4.4.1. Animals

The experiment involved male Wistar rats that were obtained from the Laboratory Animal Breeding Station (Pushchino RAS, Moscow Region). The rats were two months old and weighed 200–250 g. The animals were housed in individual polycarbonate cages (42 cm × 26 cm × 15 cm) during stress. After stress, the rats were placed in cages in groups of no more than eight animals. Normal housing conditions were observed for all animals: free access to water and food, a constant ambient temperature of 23 °C, and regulated direct light (12/12 h). All procedures with the animals were maintained in accordance with Directive 2010/63/EU of 22 September 2010. The animal study protocol was approved by the local ethical committee of V.P. Serbsky National Medical Research Centre of Psychiatry and Narcology (protocol No. 2, 17 February 2022).

#### 4.4.2. Experiment Design with Behavior

The experiment included three experimental groups of rats: control animals (*n* = 8); rats exposed to chronic stress (*n* = 8); and rats exposed to chronic stress with pharmaceutical composition “chitosan aerogel-clomipramine” intranasal treatment (*n* = 8). After the end of the chronic stress protocol, the first sucrose preference test was performed one day later. The behavioral tests were performed in the following order, with an interval of at least two days: the sucrose preference test, object recognition test, and forced swimming test.

#### 4.4.3. Distribution of Drugs in Blood and Brain Tissue

A separate step in the experiment was performed to determine the distribution of clomipramine in the blood and brains of rats. Brain and blood tissue samples were taken from the animals at the following time points after intranasal injection of the drug: 10 min, 30 min, 1 h, 2 h, and 4 h. Each time point of the experiment included samples from four individual animals. Blood was collected after decapitation, and brain sections were taken from the same rats. The blood was collected in tubes with EDTA and centrifuged at 2000× *g* for 10 min; then, plasma was collected and stored at −20 °C. After brain extraction, brain slices were made under certain conditions, which included cold. The isolated tissue samples were frozen in liquid nitrogen and then stored at −20 °C until analysis.

#### 4.4.4. Chronic Stress

The experiment involved an ultrasound model of depression-like state in rats, which was induced by the action of an ultrasound with variable wave frequency. This model consisted of the animals being alternately exposed to ultrasound frequencies in the range of 20-45 kHz continuously for three weeks. An ultrasound generator, Weitech (Wavre, Belgium), was used for this purpose. Frequency ranges were alternated and replaced every ten minutes. Short frequencies, 20–25 kHz, medium frequencies, 25–40 kHz, and high frequencies, 40–45 kHz, were used. The total duration of exposure to short and medium frequencies of ultrasound was 35%, and high frequencies, 30%. The sound pressure was 50 dB ± 5 dB (fluctuations ± 10%). During exposure to variable frequency ultrasound, the rats were housed in individual cages at a distance of 1.5 m from the ultrasound generator, with the location of the cages varying daily. The drugs were administered to the animals during chronic stress daily for three weeks [48,49,50].

#### 4.4.5. Drug Administration

**Figure 9:**
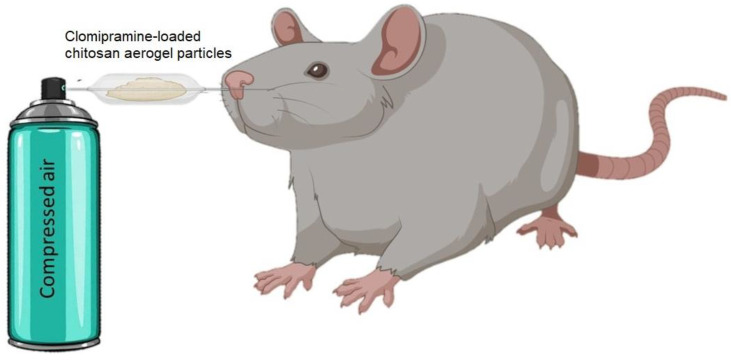
Schematic representation of the nasal administration of the pharmaceutical composition “chitosan aerogel—clomipramine” to rats.

The weight of the powdered composition for intranasal therapy, “chitosan aerogel-clomipramine”, was determined for each animal according to the body weight of the animal. The pharmaceutical composition was weighed immediately before administration to each rat separately. The dose was divided equally between the two nostrils, i.e., the drug was injected twice into each animal, once in each nostril. The following intranasal administration protocol was used: (1) the drug was poured into a cone-shaped tip, which was connected to an air supply tube from a compressed air cylinder; (2) the free end of the cone-shaped tip was placed in the rat’s nostril; (3) at the same time, air from the balloon was supplied by pressing a push-button. The scheme of the intranasal administration of the drug is illustrated in Figure 9.

The S and control groups received intranasal saline in a volume of 30 μL for 21 days. Clomipramine was administered daily at 10 am in the same dose once to study the drug distribution in the blood and brain [51].

#### 4.4.6. Sucrose Preference Test

Rats were placed in individual cages and presented with two drinkers, one with pure water and the other with 1% sucrose solution, for one day under free-choice conditions. The drinkers were swapped every six hours to eliminate site preference. After 24 h, the volume of fluid drunk from both drinkers was measured, and the sucrose preference index was calculated using the formula: Vs/(Vs + Vw) × 100%, where Vw is the volume of pure water drunk, and Vs is the volume of 1% sucrose solution drunk [49]. Rats in the normal state perceived sweetened water as more pleasant than pure water and preferred it.

#### 4.4.7. Forced Swim Test

This test was used to evaluate despair behavior, which is an indicator of a depressive-like state in rats. The test was performed in a transparent cylinder (with a diameter of 31 cm and a height of 40 cm) filled with water (at temperature of 23 °C) to the mark at the height of 30 cm from the bottom of the cylinder. The animal was placed in the cylinder with water for eight minutes. During the last six minutes, the total immobility time (immobile freezing in the water column), which is the main indicator of despair behavior, was recorded using RealTimer software (OpenScience, Moscow, Russia) [49].

#### 4.4.8. Object Recognition Test

This test was used to evaluate long-term memory. A square arena (45 cm × 45 cm × 40 cm) was used for the test. The test was performed in three stages with a 24 h break. On the first day of testing, the rat was placed in an empty arena for 5 min for habituation. On the second day, two objects of similar shape and size were placed in the arena, the rat was placed in the center of the arena, and the total time the rat explored each object was recorded for 5 min. On the third day, one of the objects in the arena was replaced by a new object, different in shape and size. The rat was placed in the center of the arena, and the total examination time of the old and new objects was recorded. In the data analysis, the total exploration time of both objects during training and the test was estimated. Animals that examined the objects during training or the test for less than 10 s were excluded; then, the discrimination index was calculated according to the formula: (Tn − To)/(Tn + To) × 100, where Tn is the total time to examine the new object at the third stage of the test, and To is the total time to examine the old object at the third stage of the test [52]. The discrimination index allowed us to evaluate the level of remembering the old object and recognizing the new one in the animals.

### 4.5. Sample Preparation for the Determination of Clomipramine in Blood Plasma and Brain Tissues

The article [53] described the sample preparation of rat blood plasma and rat brain tissue, as well as a detailed description of the detection technique. A tandem SCIEX 4500QTRAP mass spectrometer, combined with a Shimadzu 30ACMP liquid chromatograph, was used for the analysis. Chromatographic separation of the components of the test sample was carried out in RPLC mode using a chromatographic column Phenomenex Phenyl-Hexyl 2.1 × 50 mm 2.6 um. The detection conditions were chosen according to [53]: phase A (water; 5 mM Ammonium Formate); phase B (Acetonitrile; 5 mM Ammonium Formate); a flow rate of 0.45 mL/min; and a sample input volume of 1 µL.

## Figures and Tables

**Table 1:** Results of analytical studies of chitosan aerogel particles obtained by two methods.

Sample	S, m^2^/g	V_BJH_, cm^3^/g	D_pore_, nm	p_skeletal_, g/cm^3^	p_bulk_, g/cm^3^	P, %	d_av_, µm	d_aero_, µm
CS1	254	1.49	24	2.050	0.017	99.2	292	49
CS2	253	1.50	24	2.368	0.017	99.3	296	51
CS3	224	1.35	26	1.676	0.017	99.0	132	22
CS4	237	1.44	25	1.909	0.021	98.9	317	59
CH1	225	1.33	25	1.909	0.026	98.6	92	19
CH2	212	1.21	25	1.909	0.022	98.8	93	26
CH3	240	1.45	26	1.843	0.018	99.0	89	15
CH4	250	1.51	24	1.843	0.016	99.1	83	13

**Table 2:** Concentrations of all reagents in obtaining samples of chitosan aerogel particles.

C_ch_, wt.%	C_acetic acid_, M	C_NaOH_, M
1	0.1	1
1	0.2	1
1	0.2	0.1
1	0.1	0.1

## Data Availability

Data available on request.
